# Effect of *Morus alba* L. (mulberry) leaves on anxiety in mice

**DOI:** 10.4103/0253-7613.40487

**Published:** 2008

**Authors:** A.V. Yadav, L.A. Kawale, V.S. Nade

**Affiliations:** Government College of Pharmacy, Vidyanagar, Karad, Dist. Satara - 415 110, India; 1 Department of Pharmacology, N.D.M.V.P. Samaj's College of Pharmacy, Gangapur Road, Nashik - 422 002, India

**Keywords:** Anxiolytic, mice, mulberry, sedative

## Abstract

**Objective:**

The aim of the present work is to evaluate the anxiolytic effect of a methanolic extract of *Morus alba* L. leaves in mice.

**Materials and Methods:**

The hole-board test, elevated plus-maze paradigm, open field test, and light/dark paradigm were used to assess the anxiolytic activity of the methanolic extract of *M. alba* L. *Morus alba* extract (50, 100, and 200 mg/kg, i.p.) and diazepam (1 mg/kg, i.p.) were administered 30 min before the tests.

**Results:**

The results showed that the methanolic extract of *M. alba* significantly increased the number and duration of head poking in the hole-board test. In the elevated plus-maze, the extract significantly increased the exploration of the open arm in similar way to that of diazepam. At a dose of 200 mg/kg i.p. the extract significantly increased both the time spent in and the entries into the open arm by mice. Further, in the open field test, the extract significantly increased rearing, assisted rearing, and number of squares traversed, all of which are demonstrations of exploratory behavior. In the light/dark paradigm, the extract produced significant increase in time spent in the lighted box as compared to vehicle. The spontaneous locomotor activity count, measured using an actophotometer, was significantly decreased in animals pretreated with *M. alba* extract, indicating a remarkable sedative effect of the plant.

**Conclusion:**

The results of the present study suggest that a methanolic extract of *M. alba* leaves may possess an anxiolytic effect.

Anxiety affects one-eighth of the total population worldwide and has become an important area of research in psychopharmacology during this decade. Benzodiazepines (BZDs) are the major class of compounds used in anxiety and they remain the most commonly prescribed treatment for anxiety. However, the realization that BZDs have a narrow safety margin has prompted many researchers to evaluate new compounds in the hope of identifying other anxiolytic drugs with fewer unwanted side effects.[1]

The mulberry tree, a plant of the family Moraceae and the genus *Morus*, has been widely cultivated to feed silkworms. The leaves and the roots of *M. alba* have also been used in traditional medicine as a cathartic, analgesic, diuretic, antitussive, sedative, hypotensive, and antiphlogistic and for the treatment of edema.[2] The decoction of the leaves is used as a gargle for relief of inflammation of the throat. The plant contains flavonoids, coumarine, and stilbene, which have hepatoprotective and free radical scavenging activity.[3] The other uses of *M. alba* are as a hypoglycemic,[4] cardioprotective,[5] and neuroprotective agent.[6] The mulberry fruit has been used as a medicinal agent to nourish the blood and for the treatment of weakness, fatigue, anemia, and premature graying of hair. In addition, some phenolic compounds from *M. alba* have been reported to have antioxidant properties. A piperidine alkaloid and some glycoproteins were isolated from the bark and leaves, which had antidiabetic effects.[7]

Phytochemical reports on *M. alba* L. indicates that the plant contains flavonoids, tannins, triterpenes, anthocyanins, anthroquinones, phytosterols, sitosterols, benzofuran derivatives, morusimic acid, oleanolic acid, alkoloids, steroids, saponins, and phenolic compounds.[5,8] A survey of the literature on *M. alba* revealed only a few pharmacological reports on the plant. No major investigative reports were found pertaining to its CNS activity; therefore, we undertook the present study to determine the anxiolytic potential of *M. alba* by using different animal models for anxiety based on exploratory behavior.

## Materials and Methods

### Extract preparation

Fresh leaves of the plant *M. alba* L. (1 kg) were collected from an area in Nashik and authenticated at the Botanical Survey of India, Pune, where a voucher specimen of the plant was also deposited (voucher No. NVMA2). The leaves were washed and cut into pieces and air dried. The powdered plant material was defatted using petroleum ether (60-80°C) using a Soxhlet extractor. The marc was further extracted by methanol for 72 h to obtain the extract. The extract was filtered and evaporated to dryness under reduced pressure on a rotary evaporator. The yield of methanolic extract of *M. alba* L. (MAE) leaves was found to be 2.2% w/w. Before use, the extract was dissolved in distilled water for administration intraperitoneally (i.p.).

### Phytochemical screening

Phytochemical investigation of the extract for the presence of phenolic compounds, flavonoids, tannins, triterpenes, anthocyanins, anthroquinones, and sterols was carried out using the methods previously described by Kokate (1994) and Trease and Evans (1997). The presence of alkaloids and saponins was also ascertained.

### Animals

Albino male Swiss mice (18-22 g) were used for the study. The animals were housed in colony cages and maintained under standard environmental conditions: 25 ± 2°C temperature, 12:12 h light: dark cycle, and 45-55% relative humidity, with free access to food and water *ad libitum*. The animals were fasted overnight and during the experiment. All experiments were carried out during the light period (08.00-16.00 h). The Institutional Animal Ethical Committee approved the protocol of the study.

The animals were divided into five groups, each containing six mice. The groups of mice were assigned to receive one of the following: (i) vehicle (distilled water 0.1 ml/10 g of body weight, i.p.); (ii) diazepam (1 mg/kg, i.p.); (iii) MAE (50 mg/kg, i.p.); (iv) MAE (100 mg/kg, i.p.); or (v) MAE (200 mg/kg, i.p.); this group pattern was used to assess the behavioral parameters. The doses of MAE were selected on the basis of previous experiments conducted in our laboratory.

### Drugs and chemicals

Diazepam (Ranbaxy Laboratories Ltd., Mumbai) was used as the standard anxiolytic agent. Petroleum ether (60-80°C) and methanol were purchased locally and were of analytical grade. Distilled water was used as vehicle.

### Acute toxicity test

The extract was administered orally and i.p. in doses of 50, 100, 200, 500, 1000, 1500 and 2000 mg/kg to different groups of mice. The mortality rate was observed and recorded for a 24-h period.

### Behavioral parameters used to test anxiolytic activity

#### Hole-board test:

The hole-board apparatus was used as described earlier.[9] The apparatus consists of a wooden box (40 × 40 × 25 cm) with 16 holes (each of diameter 3 cm) evenly distributed on the floor. The apparatus was elevated to the height of 25 cm. Mice were treated with the MAE (50, 100 and 200 mg/kg, i.p.) or vehicle 30 min before they were placed in the apparatus. The numbers of head pokes during a 5-min period were recorded. Diazepam (1 mg/kg, i.p.), an anxiolytic agent was used as a reference drug.

#### Elevated plus-maze test (EPM):

The EPM consisted of two open arms (35 × 5 cm) crossed with two closed arms (35 × 5 × 20 cm). The arms were connected together with a central square of 5 × 5 cm. The apparatus was elevated to the height of 25 cm in a dimly illuminated room. Mice were treated with MAE (50, 100, and 200 mg/kg), diazepam (1 mg/kg, i.p), or vehicle 30 min before being placed individually in the centre of the EPM, facing a closed arm. The time spent in both the open and closed arms was recorded for 5 min. The numbers of entries into the open and closed arms were also counted during the test. An entry was defined as having all four paws within the arm.[10]

#### Open field test:

The apparatus consisted of a wooden box (60 × 60 × 30 cm). The floor of the box was divided into 16 squares (15 × 15 cm). The apparatus was illuminated with a 40-W lamp suspended 100 cm above. Mice were treated with MAE (50, 100 and 200 mg/kg) or vehicle. After 30 min, they were placed individually in one of the corner squares; the number of rearing, assisted rearing (forepaws touching the walls of the apparatus), and the number of squares crossed were counted for 5 min. Diazepam (1 mg/kg, i.p.) was used as the positive control drug.[9,11]

#### Light/dark exploration test:

The apparatus consisted of two boxes (25 × 25 × 25 cm) joined together. One box was made dark by covering its top with plywood, whereas a 40-W lamp illuminated the other box. The light source was placed 25 cm above the open box. The mice were placed individually in the centre of the lit box and observed for the next 5 min for the time spent in the lit and dark boxes. The mice were treated with MAE (50, 100, and 200 mg/kg), diazepam (1 mg/kg, i.p), or vehicle 30 min before being placed in the lit box.[12]

#### Locomotor activity:

The locomotor activity was measured using an actophotometer. The movement of the animal interrupts a beam of light falling on a photocell, at which a count was recorded and displayed digitally. Each mouse was placed individually in the actophotometer for 10 min and the basal activity score was obtained. Subsequently, the animals were divided into groups, each consisting of six animals. MAE (50, 100, and 200 mg/kg), vehicle, or diazepam (1 mg/kg, i.p) was administered and after 30 min the mice were placed again in the actophotometer for recording the activity score.[13]

### Statistical analysis

Results are expressed as mean ± S.E.M. The statistical analysis of data was done using the one-way analysis of variance (ANOVA) followed by Dunnett's test. A probability level less than 0.05 was considered statistically significant.

## Results

### Phytochemical screening

Phytochemical screening of MAE revealed the presence of phenolic compounds, flavonoids, tannins, anthocyanins, anthroquinones, sterols, alkaloids, and saponins.

### Acute toxicity test

Oral and intraperitoneal administration of MAE up to 2 gm/kg did not produce any toxic effects in mice. No mortality was observed and MAE was found to be safe at the given doses.

### Hole-board test

**Figure 1:**
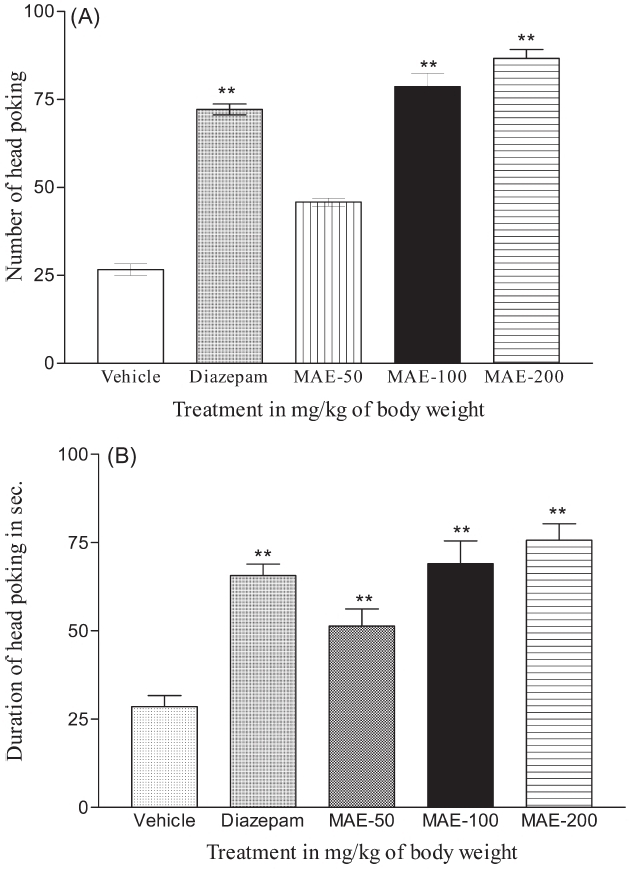
Effect of MAE on (A) number of head poking and (B) duration of head poking in mice. Each column represents mean ± SEM (*n* = 6). **P < 0.01 vs vehicle (one-way ANOVA followed by Dunnett's test)

Each mouse was placed individually in the hole-board apparatus and the number of head pokes and the duration of head poking were noted. With the dose of 50 mg/kg, i.p., of MAE there was no significant increase in number of head pokes when compared with vehicle. MAE at 100 and 200 mg/kg, i.p., increased the number of head pokes significantly (*P* < 0.01) and dose dependently. The duration of head poking was also significantly (*P* < 0.01) increased by MAE at all doses. The reference standard (diazepam, 1 mg/kg, i.p.)-treated group showed significant increase in exploratory activity (*P* < 0.01) [Figure 1A and B].

### Elevated plus-maze test

The vehicle-treated mice spent 30.8 ± 6.4 s in the open arm and 243.5 ± 8.0 s in the closed arm, with 8.3 ± 1.2 entries into the open arm and 14.7 ± 1.0 entries into the closed arm. MAE (100 and 200 mg/kg) and diazepam (1 mg/kg) induced significant (*P* < 0.01) increase in the occupancy in the open arm. MAE in the dose of 50 and 100 mg/kg did not cause a significant decrease in the time spent in the closed arm, whereas MAE at a dose of 200 mg/kg and diazepam brought about a significant (*P* < 0.01) decrease in the time spent in the closed arm. The animals treated with diazepam and MAE (200 mg/kg) showed a decreased preference for the closed arm and significantly (*P* < 0.01) increased entries into the open arm. MAE at 50 and 100 mg/kg did not produce any significant increase in open arm entries [Table 1].

**Table 1:** Effect of MAE on animals' stay in the open and enclosed arms of the elevated plus-maze in mice

*Treatment*	*Time spent in the open arm(s)*	*Time spent in the enclosed arm(s)*	*Entries into open arm*	*Entries into enclosed arm*
Vehicle	30.8 ± 6.4	243.5 ± 8.8	8.3 ± 1.26	14.7 ± 1.0
Diazepam (1 mg/kg, i.p.)	107.0 ± 12**	169.0 ± 13.1**	20.5 ± 1.9**	9.7 ± 0.9
MAE (50 mg/kg, i.p.)	36.50 ± 9.3	218.7 ± 8.5	7.3 ± 1.4	11.50 ± 1.3
MAE (100 mg/kg, i.p.)	89.8 ± 8.9**	216.8 ± 9.6	9.2 ± 1.3	10.7 ± 1.4
MAE (200 mg/kg, i.p)	117.0 ± 7.1**	177.2 ± 8.6**	17.5 ± 1.7**	10.5 ± 1.0

*n* = 6;

** *P* < 0.01 *vs* vehicle (one-way ANOVA followed by Dunnett's test)

### Open field test

The vehicle-treated mice traversed 89.0 ± 4.7 squares and showed 11.5 ± 1.2 assisted rearing and 4.00 ± 1.0 self-rearing during the test interval of 5 min. MAE at 50, 100, and 200 mg/kg and diazepam brought about a significant (*P* < 0.01) and dose-dependant increase in the number of squares traversed. The assisted rearing and self rearing was significantly (*P* < 0.05 and *P* < 0.01, respectively) increased by MAE (100 and 200 mg/kg) and diazepam; MAE at 50 mg/kg did not a produce significant effect [Table 2].

**Table 2:** Effect of MAE on rearing and locomotion in open field test model

*Treatment*	*Rearing*	*Assisted rearing*	*No. of square traversed*
Vehicle	4.0 ± 1.0	11.5 ± 1.2	89.0 ± 4.7
Diazepam (1 mg/kg, i.p.)	16.0 ± 1.5**	21.7 ± 3.3**	169.5 ± 7.9**
MAE (50 mg/kg, i.p.)	12.0 ± 1.2	13.2 ± 1.2	125.2 ± 7.5**
MAE (100 mg/kg, i.p.)	22.5 ± 4.5**	20.5 ± 1.8*	140.5 ± 8.2**
MAE (200 mg/kg, i.p)	30.3 ± 2.5**	20.8 ± 2.0*	158.7 ± 8.1**

*n* = 6;

* *P* < 0.05,

** *P* < 0.01 *vs* vehicle (one-way ANOVA followed by Dunnett's test)

### Light/dark exploration test

The animals treated with MAE (100 and 200 mg/kg) and diazepam (1 mg/kg) showed significant (*P* < 0.05 and *P* < 0.01) increase in the time spent in the lighted box and decrease in the time spent in the dark box. All the treatments failed to produce any significant change in the number of crossings and the transfer latency. The extract at a dose of 50 mg/kg did not produce any significant change in any of the parameters [Table 3].

**Table 3:** Effect of MAE on time spent, number of crossing, and transfer latency in light/dark exploration model

*Treatment*	*Time spent in lighted box (s)*	*Time spent in dark box (s)*	*No. of crossings*	*Transfer latency (s)*
Vehicle	80.0 ± 8.4	205.7 ± 6.4	17.3 ± 1.5	15.8 ± 2.1
Diazepam (1 mg/kg, i.p.)	167.5 ± 8.6**	124.2 ± 8.6**	19.8 ± 2.1	20.7 ± 3.3
MAE (50 mg/kg, i.p.)	81.8 ± 6.3	205.3 ± 6.5	16.3 ± 2.1	19.5 ± 2.1
MAE (100 mg/kg, i.p.)	139.0 ± 16.5**	155.7 ± 16.3*	14.2 ± 1.0	46.8 ± 19.6
MAE (200 mg/kg, i.p)	131.2 ± 14.3*	155.5 ± 13.2*	8.5 ± 1.9*	36.0 ± 16.7

*n* = 6;

* *P* < 0.05,

** *P* < 0.01 *vs* vehicle (one-way ANOVA followed by Dunnett's test)

### Locomotor activity

MAE in doses of 100 and 200 mg/kg produced significant (*P* < 0.01) reduction in locomotor activity as compared to the control animals. The diazepam-treated group also revealed a statistically significant decrease in locomotor activity (*P* < 0.01) [Table 4].

**Table 4:** Effect of MAE on locomotor activity in mice, assessed using the actophotometer

*Treatment*	*Locomotor activity (score) in 10 min*		*Reduction in activity (%)*
	Before	After treatment	
Vehicle	707.8 ± 40.2	652.3 ± 46.5	8.0 ± 2.6
Diazepam (1 mg/kg, i.p.)	697.3 ± 47.4	343.0 ± 27.4	50.0 ± 4.6**
MAE (50 mg/kg, i.p.)	475.5 ± 50.5	377.7 ± 49.5	21.0 ± 4.4
MAE (100 mg/kg, i.p.)	757.3 ± 73.8	433.7 ± 30.9	41.9 ± 2.4**
MAE (200 mg/kg, i.p)	572.5 ± 70.9	264.2 ± 48.9	55.1 ± 5.5**

*n* = 6;

** *P* < 0.01 *vs* vehicle (one-way ANOVA followed by Dunnett's test)

## Discussion

The etiology of most anxiety disorders is not yet fully understood, but the picture has become a little clearer in the recent past. The benzodiazepines (BZDs) are relatively safe and are widely used anxiolytic agents. These agents are known to act through the BZD-GABA receptors. The role of GABA in anxiety is well established.[14]

Despite the widespread traditional use of *M. alba* for treating various disorders, there are no reports of any scientific evaluation of its pharmacological effects. The present work demonstrated that the methanolic extract of *M. alba* had anxiolytic effects in mice in several behavioral parameters, like the hole-board, elevated plus-maze, open field, and light/dark paradigms. The anxiolytic activity of some agents have been assessed by using the hole-board test.[11] A significant increase in the exploratory head-dipping behavior was observed after treatment with 100 and 200 mg/kg of *M. alba* extract, thus reinforcing the hypothesis that it has anxiolytic-like activity.

The EPM is one of most popular animal tests for research on behavioral pharmacology of anxiety. It involves spontaneous or natural aversive stimuli, i.e., height, unprotected opening, and novelty.[15] Several plants that are used in folk medicine to diminish anxiety are reported to bring about an increase in the exploration of the open arms in the EPM test.[16] In EPM, naïve mice will normally prefer to spend much of their allotted time in the closed arms. This preference appears to reflect an aversion towards open arms that is generated by fear of open spaces. Drugs that increase open arm exploration are considered as anxiolytics and the reverse holds true for anxiogenics.[17] In our study, we observed that MAE (100 and 200 mg/kg) induced significant increases in the both the number of entries and time spent in the open arms. The number of entries and the time spent in the closed arms were reduced in the extract-treated group as compared to the control group. The results obtained in the open field test showed that MAE administration significantly increased rearing, assisted rearing, and number of squares traversed, which supports the anxiolytic-like activity of MAE.

The light and dark paradigm is based on the natural aversion of mice to brightly lit places. Anxiolytics reduce the natural aversion to light and increase the time spent in the lit compartment. In this model, compared to vehicle, MAE produced significant increase in the time spent in the lighted box and decrease in the time spent in the dark box, thus demonstrating its anxiolytic-like activity.

Locomotor activity is considered as an index of alertness, and a decrease indicates a sedative effect.[16] The extract was able to induce a motor depressant effect, indicating a significant skeletal muscle relaxant and sedative effect of the plant.[10]

The anxiolytic, anticonvulsant, muscle relaxant, and sedative-hypnotic actions of the BZDs make them the most important GABA_A_-modulating drugs. The mechanism of anxiolytic action of MAE might involve an action on GABAergic transmission; however, further studies are needed to ascertain this.

Earlier reports on the chemical constituents of plants and their pharmacology suggest that plants containing flavonoids, saponins, and tannins possess activity against many CNS disorders.[11] Photochemical tests of MAE revealed the presence of flavonoids, tannins, and saponins. It is possible that the mechanism of anxiolytic action of MAE could be due to the binding of any of these phytochemicals to the GABA_A_-BZD complex. In support of this, it has been found that flavones, which are present in *M. alba*, bind with high affinity to the BZD site of the GABA_A_ receptor.[10]

The results obtained in this study suggest that the extract of the leaves of *M. alba* possesses anxiolytic and muscle-relaxant activities, which is possibly mediated through the GABA_A_-BZD mechanism. Thus, *M. alba* L. has potential clinical application in the management of anxiety and muscle tension disorders. Further investigation of the mechanism(s) of action of the plant extract, as well as the active substance(s) responsible for its biological actions, is necessary.
